# Hypochlorous Acid-Induced Heme Degradation from Lactoperoxidase as a Novel Mechanism of Free Iron Release and Tissue Injury in Inflammatory Diseases

**DOI:** 10.1371/journal.pone.0027641

**Published:** 2011-11-22

**Authors:** Carlos Eduardo A. Souza, Dhiman Maitra, Ghassan M. Saed, Michael P. Diamond, Arlindo A. Moura, Subramaniam Pennathur, Husam M. Abu-Soud

**Affiliations:** 1 Department of Obstetrics and Gynecology, The C.S. Mott Center for Human Growth and Development, Wayne State University School of Medicine, Detroit, Michigan, United States of America; 2 Federal University of Ceará, Fortaleza, Ceará, Brazil; 3 Division of Nephrology, Department of Internal Medicine, University of Michigan Medical School, Ann Arbor, Michigan, United States of America; 4 Department of Biochemistry and Molecular Biology, Wayne State University School of Medicine, Detroit, Michigan, United States of America; University of Giessen Lung Center, Germany

## Abstract

Lactoperoxidase (LPO) is the major consumer of hydrogen peroxide (H_2_O_2_) in the airways through its ability to oxidize thiocyanate (SCN^−^) to produce hypothiocyanous acid, an antimicrobial agent. In nasal inflammatory diseases, such as cystic fibrosis, both LPO and myeloperoxidase (MPO), another mammalian peroxidase secreted by neutrophils, are known to co-localize. The aim of this study was to assess the interaction of LPO and hypochlorous acid (HOCl), the final product of MPO. Our rapid kinetic measurements revealed that HOCl binds rapidly and reversibly to LPO-Fe(III) to form the LPO-Fe(III)-OCl complex, which in turn decayed irreversibly to LPO Compound II through the formation of Compound I. The decay rate constant of Compound II decreased with increasing HOCl concentration with an inflection point at 100 µM HOCl, after which the decay rate increased. This point of inflection is the critical concentration of HOCl beyond which HOCl switches its role, from mediating destabilization of LPO Compound II to LPO heme destruction. Lactoperoxidase heme destruction was associated with protein aggregation, free iron release, and formation of a number of fluorescent heme degradation products. Similar results were obtained when LPO-Fe(II)-O_2_, Compound III, was exposed to HOCl. Heme destruction can be partially or completely prevented in the presence of SCN^−^. On the basis of the present results we concluded that a complex bi-directional relationship exists between LPO activity and HOCl levels at sites of inflammation; LPO serve as a catalytic sink for HOCl, while HOCl serves to modulate LPO catalytic activity, bioavailability, and function.

## Introduction

Myeloperoxidase (MPO) and lactoperoxidase (LPO) are homologous members of the mammalian peroxidase superfamily which also include eosinophil peroxidase and thyroid peroxidase. These enzymes share an overall 60–70% amino acid sequence homology [1]–[3]. The heme moiety in the peroxidases is attached to the enzymes through an imidazole nitrogen; its main function is to catalyze the H_2_O_2_-dependent oxidation of halides and pseudo halides to generate the corresponding hypohalous acid, in the catalysis of oxidative reactions [4]–[6]. LPO is a monomeric protein with a single polypeptide chain of 78.5 kDa, and uses the pseudo halide, thiocyanate (SCN^−^) as a preferred substrate to generate hypothiocyanous acid (HOSCN) [6], [7]. Whereas, MPO is a 150–165 kDa, homodimer, each subunit comprising of a pair of light and heavy chains, and uses chloride (Cl^−^) as the preferred substrate to generate hypochlorous acid (HOCl) [2], [3], [8]. The reaction of peroxidases and the co-substrate H_2_O_2_ involves oxygen transfer to Fe(III) to form a ferryl porphyrin π cation radical (Fe(IV) = O^+π•^; Compound I) intermediate as an initial step in the classic peroxidase catalytic cycle [2], [3], [6], [9]. Compound I is a short-lived intermediate and is readily reduced to its 1 electron equivalent, forming a ferryl intermediate (LPO-Fe(IV) = O; Compound II) a longer-lived intermediate whose decay to ferric state is considered to be the rate-limiting step during steady-state catalysis [2], [3], [10].

Enhancements in peroxidase catalysis due to reduction of compounds I and II have been noted with a series of organic and inorganic substrates [10]–[13] and physiological reductants generating cytotoxic oxidants and diffusible radical species [14], [15]. In addition, the heme moiety of mammalian peroxidases can accommodate a large variety of molecules as a ligand of the iron cation, in which their bindings to the heme moiety inhibit the catalytic activity of the enzymes [10]–[13]. For example, superoxide and O_2_ can serve as ligands for Fe(III) and Fe(II) of peroxidases, respectively, to generate ferrous dioxy complex (Fe(II)-O_2_ complex), Compound III [16]–[18].

Hypochlorous acid (HOCl) generated by MPO plays an important role in killing microorganisms [19]. However, it is also capable of initiating lipid peroxidation, promoting an array of posttranslational modification of target proteins, injure normal tissues by bleaching the heme moieties of hemoproteins, and oxidatively destroying electron transport chains [20]–[22]. The rate of HOCl production by neutrophils has been shown to be as high as 450 µM/h in an in vitro study, which may be less in an *in vivo* model, due to scavenging actions of antioxidants [23], [24]. Recent studies have shown that catalytically active MPO and its oxidative species are present in human atherosclerotic lesions [25]–[27]. This implicates the enzyme involvement in low-density lipoprotein oxidation *in vivo* [28]. It has also been shown that iron accumulates in atherosclerotic lesions in a catalytically active form [29], [30]. The source of this iron is still unclear, but it is thought to result from hemoglobin released from damaged red cells at sites of vascular turbulence or in hemorrhagic atheromatous plaques [31]. Recently we have shown that under oxidative stress, MPO may serve as a source of free iron through a mechanism that involves heme depletion [32].

LPO and MPO, and their final products commonly participate in tissue injury in a large number of inflammatory conditions, and are used as markers for lung and atherosclerotic cardiovascular diseases [1], [2], [33], [34]. To further assess the potential physiological relevance of HOCl interactions with LPO under conditions that more closely mirror high-risk subjects, such as lung and heart diseases, we investigated the effect of varying HOCl concentrations on LPO-Fe(III) and LPO-Fe(II)-O_2_ catalysis in the absence and presence of plasma levels of SCN^−^. A variety of analytical techniques including optical absorbance spectrophotometry, rapid kinetics measurements, high performance liquid chromatography (HPLC), free iron, and SDS-PAGE were utilized in this study. Our results revealed that low levels of HOCl binding to LPO-Fe(III) resulted in a rapid formation and decay of LPO Compounds I and II, suggesting that LPO may serve as a catalytic sink for the removal of HOCl. Increase in the levels of HOCl cause rapid accumulation of LPO Compound II followed by heme destruction and subsequent iron release and protein aggregation. Heme destruction and protein aggregation can be partially or completely prevented in the presence of increasing levels of SCN^−^.

## Results

### Spectroscopic and rapid-kinetics characterization of the interaction between HOCl and LPO-Fe(III)

**Figure 1:**
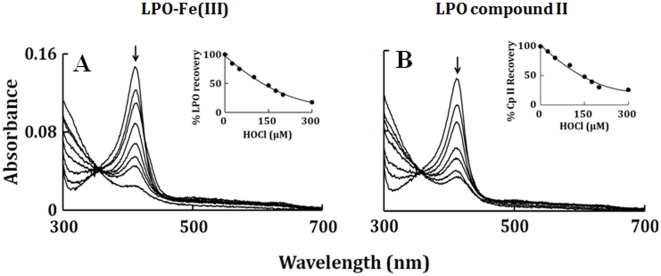
Optical absorbance spectra for concentration dependent of HOCl-mediated heme depletion of LPO-Fe(III) and LPO Compound II. Spectral traces were recorded after 2 h of incubation of a fixed amount (1.5 µM) of LPO-Fe(III) (Panel A) and LPO Compound II (Panel B) with increasing concentration of HOCl (0, 25, 50, 100, 150, 175, 200 and 300 µM), at 25°C. Arrows in Panel A and B indicate the direction of spectral change. The LPO recovery estimated from the absorbance values at 432 from each spectral scan recorded in the Panels A and B are plotted versus HOCl concentration (Panel A and B insets). These data are representative of three independent experiments.

**Figure 2:**
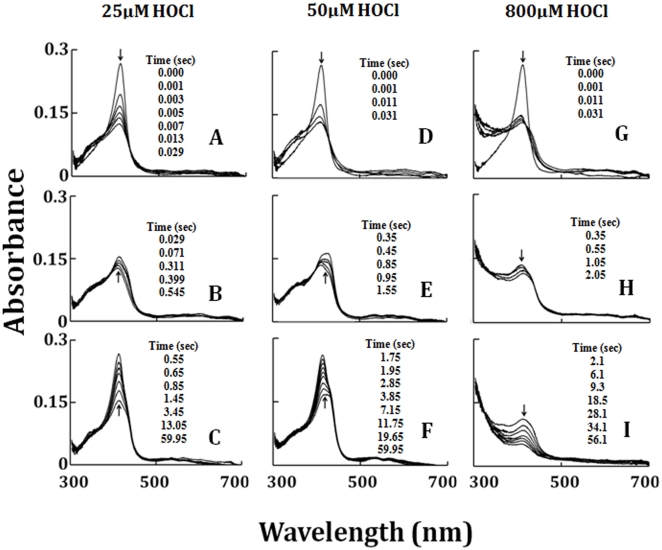
Formation of LPO Compound I and II and their exhaustion through the reactions of LPO-Fe(II) with increasing concentration of HOCl. Rapid-scanning diode array spectra were recorded during the reaction of LPO-Fe(III) (3 µM) with a buffer solution containing 25 µM (Panels A, B, and C), 50 µM (Panels D, E, and F), and 800 µM (Panels and G, H, and I) HOCl, at 10°C. Selected spectra were omitted from each panel for clarity. Arrows in the panels indicate the direction of spectral change over time. The time of each collected spectrum after initiation of the reaction is indicated in seconds. The data are representative of three independent experiments.

Spectroscopic studies demonstrated that incubation of LPO-Fe(III) and LPO Compound II with increasing concentration of HOCl for 30 minutes caused LPO heme destruction, as judged by a decrease and shift in the Soret peak at 412 and 432 nm, respectively (Fig. 1 A and B). Fig. 1 insets show the percentage recovery of LPO as a function of HOCl. We next utilized the diode array stopped-flow instrument to investigate how HOCl influences the LPO catalytic activity and function as may occur under inflammatory condition. Rapid mixing of a buffered solution supplemented with 6 µM LPO-Fe(III) with an equal volume of a similar solution supplemented with 25 µM HOCl in the absence of co-substrates resulted in the rapid formation of a transient complex that displayed a Soret absorbance at 410 nm (Fig. 2A). This spectrum differs from that of ferric LPO, whose Soret maxima is centered at 412 nm. The spectrum of the intermediate initially formed following addition of HOCl to LPO-Fe(III) is consistent with formation of LPO Compound I [35]. This LPO intermediate was formed within 30 ms after mixing at 10°C, but was unstable and rapidly converted partially into a more stable intermediate within 0.5 s, as characterized by a partial time-dependent shift of the Soret band at 412 to 432 nm, together with slight modification in the visible region from 500 to 700 nm. These spectral changes are consistent with the partial formation of LPO Compound II, as shown in Fig. 2B. LPO Compounds I and II were unstable and converted gradually to the LPO-Fe(III), within 1 minute of initiating the reaction (Fig. 2C). Rapid mixing of a solution of LPO-Fe(III) with an equal volume of a 50 µM of HOCl show similar results except that formation of Compound I (Fig. 2D) is much faster and completely converted to Compound II (Fig. 2E), which then decays immediately to LPO-Fe(III) (Fig. 2F). Rapid mixing of a solution of LPO-Fe(III) with higher HOCl concentrations (e.g. 800 µM) shows faster formation of Compound I (Fig. 2G), which is converted completely to Compound II in the next 2 s (Fig. 2H). Formation of Compound II by HOCl was accompanied by a marked decrease and flattening in the Soret absorbance region within a minute of initiating the reaction (Fig. 2I), suggesting heme degradation. These spectral changes may also suggest that, under these conditions, the majority of LPO was converted to Fe(IV) = O complex before heme destruction.

**Figure 3:**
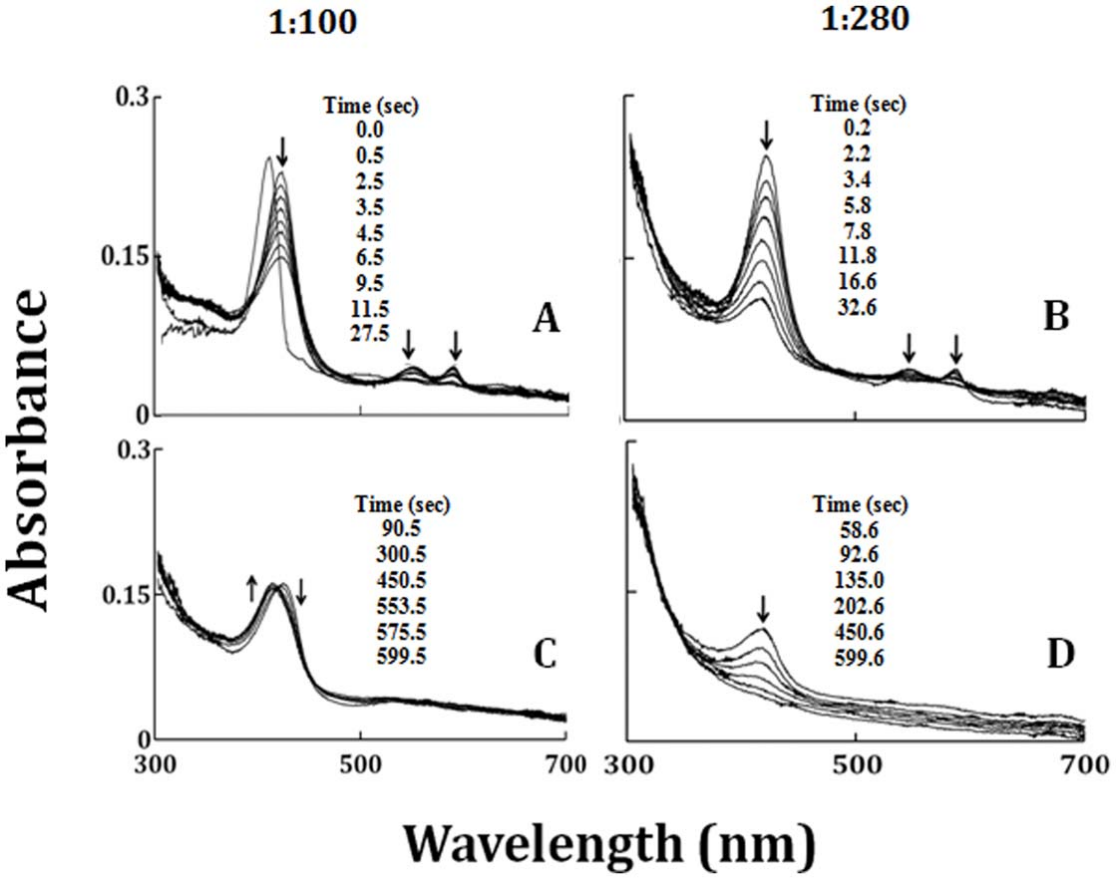
Diode array spectral changes for the reaction of LPO compound III with increasing concentration of HOCl. Rapid-scanning diode array spectra were recorded during the reaction of LPO Compound III (3 µM) with a buffer solution containing 300 µM (Panels A and B) and 840 µM (Panels and G, H, and I) HOCl, at 10°C. Arrows in the panels indicate the direction of spectral changes over time. The time of each collected spectrum after initiation of the reaction is indicated in seconds. The dashed line spectrum is the spectra of the LPO-Fe(III). The first spectrum corresponds with the initial formation of Compound III, which was generated by mixing LPO-Fe(III) with 200 µM H_2_O_2_. The experiments shown are representative of three.

To determine the role of the LPO-Fe(II)-O_2_ complex in catalytic activity, as well as to further our understanding of the potential role of LPO in catalase-like function, the direct reaction between LPO-Fe(II)-O_2_ with HOCl was carried out using rapid kinetic measurements. As shown in Figs. 3A, addition of a slight molar excess of H_2_O_2_ to LPO-Fe(III) caused immediate LPO Compound III formation, as judged by a shift in the Soret absorption peak from 412 to 424 nm, and the appearance of additional absorbance peaks in the visible range at 561 and 595 nm (17,38). This intermediate is relatively stable. Rapid mixing of a buffer solution supplemented with 6 µM LPO-Fe(II)-O_2_ against a buffer solution supplemented with 300 µM of HOCl in the absence of co-substrates (Figs. 3A,B) led to the accumulation of Compound II within the first 28 s of initiating the reaction via the transient initial formation of Compound I. Compound II that accumulated during the reaction then decay back to LPO-Fe(III) within the next 600 s (Fig. 2B). Similar behavior was observed when LPO-Fe(II)-O_2_ solution was mixed with 800 µM HOCl, except that compound II exhaustion occurred through a heme destruction pathway (Fig. 3D,E). At all HOCl concentrations tested, spectral transitions between each intermediate formed revealed distinct and well-defined isosbestic points (Figs. 2 and 3). Thus sequential formation and decay of LPO intermediates within the peroxidase cycle occur at sufficiently different rates to enable each process to be studied by conventional (i.e. single mixing) stopped-flow methods.

**Figure 4:**
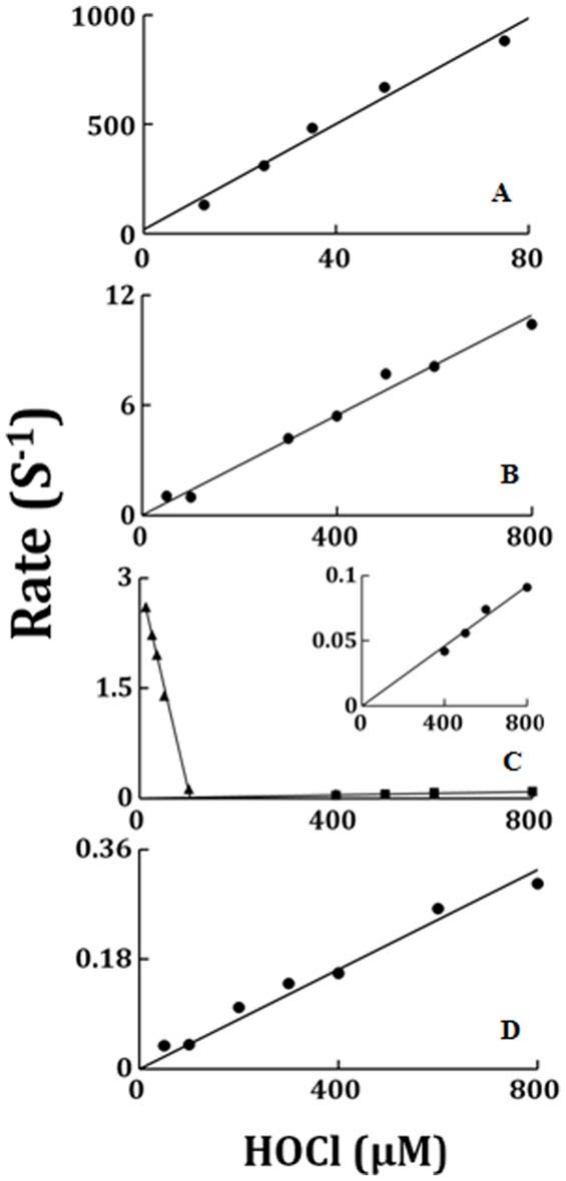
Plot of observed rate constants of various intermediates that formed upon mixing of LPO-Fe(III) or LPO-Fe(II)-O_2_ against increasing concentration of HOCl. The observed rates of LPO-Fe(III)-OCl formation monitored at 432 nm (Panel A); Compound II accumulation (Panel B); and Compound II decay(Panel C); as for Fig. 2, were plotted as a function of HOCl concentration. In Panel C, the close triangle represent kinetic parameters for Compound II decay to LPO-Fe(III), while the close circles represent kinetic parameters for Compound II destruction. The inset shows the rate of heme destruction, the curve was extrapolated to zero. Panel C is the plots of the observed rates for the LPO Compound III heme destruction versus HOCl concentration, monitored at 424 nm. The data are representative of three separate experiments.

We next utilized stopped-flow spectroscopy to investigate how HOCl interacts with catalytic intermediates of LPO. The influence of HOCl on the kinetics of LPO Compound II build-up, duration and decay were examined following rapid mixing of enzyme and various concentrations of HOCl. The time courses for the formation and decay of Compounds I and II of LPO in the absence of SCN^−^ were detected by monitoring the absorbance change at 412 and at 430 nm (data not shown). At all HOCl concentration tested, there was a monophasic decrease in absorbance at 412 nm, attributed to buildup of LPO-Fe(III)-OCl complex formation, followed by a slower, essentially monophasic decrease attributed to heme destruction. As shown in Fig. 4, Panel A, the plot of the observed rate (k_obs_) versus HOCl concentration was linear, with positive intercept indicating that the reaction is reversible in nature yielding combination and dissociation rate constants estimated from the slope and intercept of 12.1 µM^−1^ s^−1^ and 18 s^−1^, respectively. The subsequent increases in absorbance at 432 nm, attributed to Compound II formation, were also best fitted to a single exponential function. The plot of the observed rate constant as a function of HOCl concentration was linear with y-intercept close to zero and yielded a second order rate constant of 0.014 µM^−1^ s^−1^ (Fig. 4B).

At lower HOCl concentration, HOCl accelerated the decay of Compound II to LPO-Fe(III) (Fig. 2). The rate constant for decay of Compound II decreased with increasing HOCl concentration with an inflection point at 100 µM HOCl, after which the decay rate increased (Fig. 4C). This point of inflection is the critical concentration of HOCl beyond which HOCl switches its role, from mediating destabilization of LPO Compound II to LPO heme destruction. At HOCl higher than the inflection point, the rate of LPO Compound II destruction increased in a linear manner as a function of HOCl concentration line extrapolation was close to zero (Fig. 4C inset; dashed line), indicating that the heme destruction is an irreversible process. The second order rate of HOCl-mediated heme destruction, estimated from the slope (Fig. 4C inset), yielding a second order rate constant of 1×10^−4^ µM^−1^ s^−1^. Finally, HOCl significantly accelerated the rate of LPO-Fe(II)-O_2_ decay in a concentration-dependent fashion. Plots of HOCl concentration vs. observed rates of LPO-Fe(II)-O_2_ destruction demonstrated linear kinetics and yielded second order rate constants of 4×10^−4^ µM^−1^ s^−1^ (Fig. 4D).

### SCN^−^ modulates HOCl-mediated LPO heme degradation

**Figure 5:**
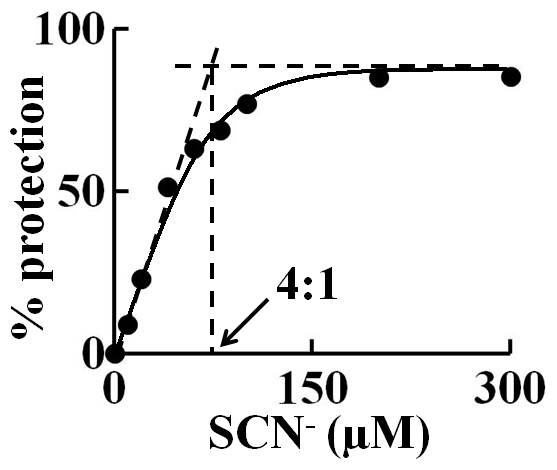
SCN^−^ prevents HOCl mediated LPO heme degradation. Fixed amount of LPO (1.5 µM) was incubated with increasing concentrations of SCN^−^ (0–300 µM) and the reaction mixtures then treated with fixed amount of HOCl (300 µM). After incubating the reaction for 2 hours, absorbance spectra were collected. The percentage protection in LPO heme content was calculated from the absorbance at 412 nm and plotted against SCN^−^ concentration. The inflection point (which occurs at 4∶1 HOCl∶SCN^−^ ratio) is marked by the dashed line with arrow. The data are averages of three independent experiments.

To test the ability of SCN^−^ in modulating HOCl-mediated LPO heme degradation, in a 1 ml (final volume) phosphate buffer solution, we first mixed a fixed amount of LPO (1.5 µM) with increasing concentration of SCN^−^ (0 to 300 µM) and then the reaction mixtures received fixed concentration of HOCl (300 µM). After 2 hours incubation, the full absorbance spectrum (from 300–700 nm) for each solution mixture was collected, and the percentage recovery in the LPO Soret absorbance peak was plotted as a function of SCN^−^ concentration. The percentage recovery increased linearly and maximized (∼85%) at 75 µM SCN^−^, a ratio of 4∶1 HOCl∶SCN^−^ (Fig. 5).

### HOCl-mediated heme destruction, protein aggregation and free iron release in LPO

**Figure 6:**
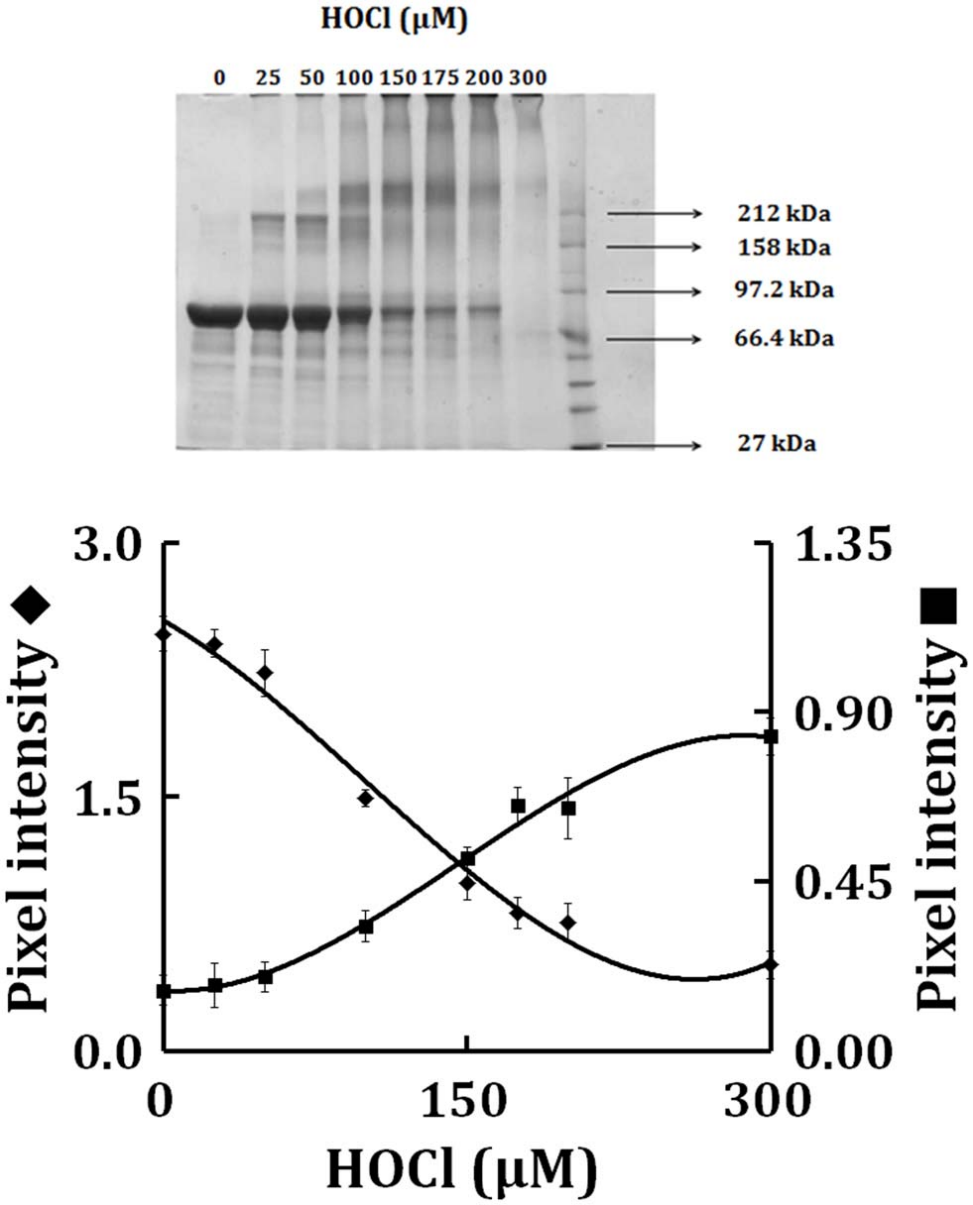
Protein aggregation of LPO treated with different HOCl concentrations. Upper panel depicts the reducing SDS-PAGE of LPO treated with HOCl, showing a decrease in the main LPO band, and a concomitant increase of high molecular weight aggregates. Lower panel displays a plot showing the variations of pixel intensity of LPO monomer (closed diagonal) and high molecular weight aggregates (closed square) as a function of HOCl concentration. Reaction was carried-out by mixing a buffered solution of LPO (1.5 µM final) with HOCl, and the gel was run after 60 minutes incubation, at 25°C. These data are representative of three independent experiments.

**Figure 7:**
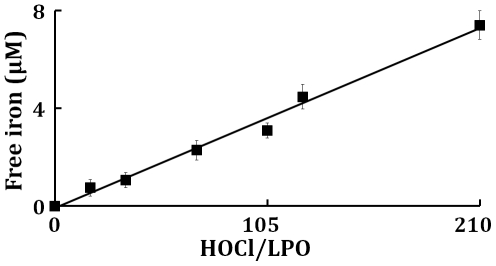
Release of free iron from LPO following treatment with HOCl. 10 µM of LPO-Fe(III) were incubated with increasing HOCl concentrations (0, 175, 350, 700, 1050, 1400, and 2100 µM) for 30 minutes, at 25°C. Free iron was assayed colorimetrically using ferrozine (for details see *Materials and Methods* section). Experiments were carried out in sodium phosphate buffer (0.2 M at pH 7.0). These data are representative of three independent experiments.

As HOCl is thought to oxidize the heme moiety of LPO, we examined whether these spectral transformations that are apparent from our UV–visible spectral analysis may represent the oxidation, free iron release, and subsequently protein aggregation. To investigate how the flattening in the Soret absorbance peak at 412 nm, in HOCl treated samples, is related to modification in the protein moiety of LPO after HOCl treatment, using reducing SDS-PAGE (Fig. 6), we analyzed the protein aggregation in LPO (1.5 µM final) after treatment with 25 to 300 µM of HOCl, by SDS-PAGE. The LPO monomer band intensity decreases slightly until quite near the 1∶100 LPO∶HOCl ratio, followed by steep plunge till 1∶200 LPO∶HOCl ratio after which it saturates. In contrast, the monomer∶oligomer band intensity showed a steady increase till 1∶200 LPO∶HOCl ratio after which it saturates. With the largest amounts of HOCl, the distinct pattern of aggregation disappeared, producing smears over the entire length of the lanes. This could be attributed either to the formation of aggregates that were too large to enter the separating gel, or to fragmentation. Bonini *et al.,* have obtained similar aggregation patterns when catalase was treated with HOCl (39). Free iron accumulation when assayed by ferrozine (as detailed in *Materials and Methods*) showed a linear increase as a function of HOCl concentration (Fig. 7).

### HPLC analysis of heme degradation products from LPO

**Figure 8:**
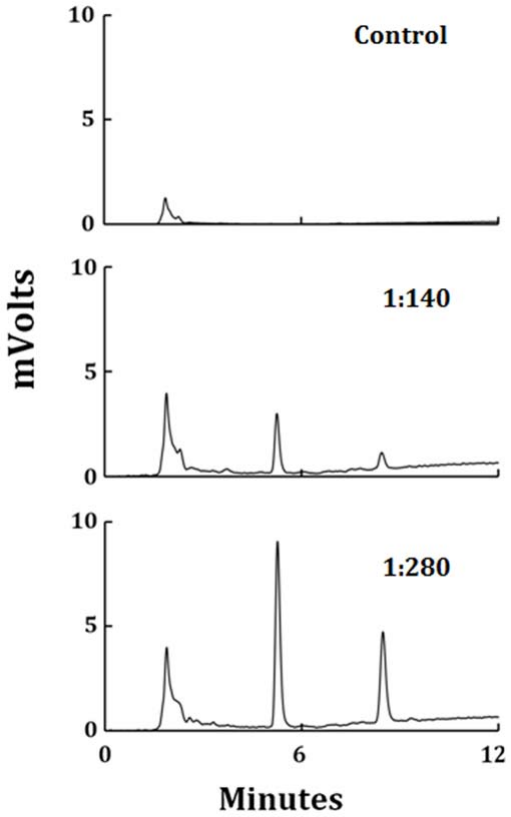
Treatment of LPO with HOCl leads to the generation of fluorescent heme degradation products. LPO (1.5 µM) was treated with increasing ratios of HOCl (control, 1∶140. and 1∶280) and analyzed by HPLC, as detailed in the *Materials and Methods* section. The fluorescent detector was set at excitation 321 nm and emission 465 nm. The molar ratio of the LPO∶HOCl used for treatment is mentioned in inset of each panel. This data are representative of three independent experiments.

Heme by itself does not have any intrinsic fluorescence, but porphyrin derivatives generated due to oxidative fragmentation of heme do have an intrinsic fluorescence. We used this property to analyze the heme fragmentation pattern after HOCl treatment of LPO. Based on a previously published report we chose to monitor the chromatograms at excitation 321 nm and emission 465 nm [36], [37]. Fig. 8 shows the chromatograms when LPO was treated with different molar ratios of HOCl. We incubated a fixed amount of LPO (10 µM) with increasing molar ratios of HOCl (1∶25, 1∶200 and 1∶400). When LPO was reacted with HOCl, there was a progressive accumulation of new heme degradation products (as a function of HOCl concentration) eluting at earlier time. By comparing the chromatograms we concluded that HOCl treatment led to the formation of at least three different fluorescent degradation products with retention times of 2, 3.3, 5 and 8 minutes, respectively. The appearance of new earlier eluting peaks in the chromatograms could be due to the formation of degradation products with decreasing hydrophobicity generated by fragmentation of the tetrapyrrole ring of the heme.

## Discussion

LPO is present in different biological fluids and epithelial surfaces, including the oral cavity and the airways [38]–[40], as part of the innate defense system. In the human oral cavity, the LPO defense system usually works in concert with other local defenses, like MPO, lysozyme and lactoferrin, to ensure tissue protection [40], [41]. Although LPO and MPO function to keep sterility of the airway system in the setting of constant exposure to inhaled debris and potential pathogens, they may also play a role in tissue defense against oxidative stress through their ability to scavenge excess H_2_O_2_ and protect the airway epithelium from its toxicity [38], [42]. This is especially important in the airways, where catalase is present in the peroxisomal system, but not secreted in the luminal fluid [43], thereby making mammalian peroxidases the main H_2_O_2_ scavengers. In addition to higher levels of LPO and MPO, patients with asthma, cystic fibrosis, and chronic obstructive pulmonary disease display higher levels of free iron in their lung compared to normal subjects [44]–[46]. As yet the source of the free iron is still unclear, but one major source of free iron may be the destruction of heme moiety from hemoproteins and the release of the iron residing in active sites of these proteins. Evidence for the involvement of the LPO/HOCl system as this source is the high affinity of LPO-Fe(III) towards HOCl and the ability of HOCl to destroy the catalytic center of the enzyme, which is, in this case, the heme moiety.

Previous study by Furtmuller *et al*., has shown that the formation of LPO compound I through the reaction of LPO-Fe(III) with HOCl is extremely fast in contrast to a two electrons reduction of H_2_O_2_, and occurs in a concentration dependent fashion [47]. The results of the present study confirmed and extended these findings, and demonstrate that HOCl not only mediates the formation of LPO Compound I, but it also mediates LPO protein aggregation, heme destruction, and subsequent iron release. During continuous monitoring the reaction of OCl^−^ with LPO-Fe(III) with diode array stopped-flow methods, rates of LPO Compound I formation were dramatically accelerated as a function of HOCl concentration. HOCl-dependent formation of Compound I was catalytic, since low levels of OCl^−^ were required, relative to the concentration of LPO used. Furthermore, HOCl-dependent formation of LPO Compound I occurred in concentrations of HOCl that span both the physiological and pathophysiological range. It is possible that the heterolytic cleavage of the O-Cl bond in an LPO-Fe(III)-OCl intermediate preferentially occur at neutral conditions, to degrade HOCl and form a ferryl porphyrin radical cation LPO-Fe(IV) = O^•^ intermediate. This intermediate is highly unstable and immediately decays to form LPO-Fe(IV) = O complex. The degree of Compound II accumulation, stability, and its exhaustion pathway depend mainly on the HOCl concentration used. The transition of the pathway of Compound II exhaustion is reflected by the deflection in the decay rate with a HOCl critical concentration of 100 µM (Fig. 3C). Below this concentration, HOCl is capable of destabilizing LPO Compound II and did not cause LPO heme destruction. Concentrations of HOCl generated by the activated blood neutrophils range between 150–425 µM HOCl per h [23], [48]. Thus, the beneficiary role of LPO is not only limited to scavenging of HOCl, but also activating LPO by accelerating the conversion of Compounds II and III to LPO-Fe(III), the active form of the enzyme. These finding may display an important application in biological systems, since scavenging HOCl by LPO may protect the airway epithelium from its toxicity. But above the critical concentration, HOCl irreversibly mediates heme destruction and subsequent free iron liberation as well as protein aggregation. Consistent with this notion, the colorimetric ferrozine-based assay for quantitation of free iron showed a corresponding increase in free iron production as a function of HOCl concentration. The toxic effect of free iron is due to its ability to generate other ROS, such as the O_2_^•−^, H_2_O_2_, and the hydroxyl radical (^•^OH), that mediate cellular mitochondria poisoning, lipid peroxidation, and oxidative phosphorylation uncoupling [49]–[51]. Free iron damages blood vessels and induces vasodilation with increased vascular permeability, leading to hypotension and metabolic acidosis [31], [52]. Under many pathological conditions such as atherosclerosis, endometriosis, and cancer, where MPO has been known to play a role, there have been reports of significant free iron accumulation [53], [54].

Compound II is the major inactive intermediate that accumulates prior to heme destruction. It is not clear whether the formation of LPO Compound II is essential for HOCl-mediated heme destruction directly or else possibly indirectly after incorporation into another intermediate, more susceptible to heme destruction than Compound II. The high stability of Compound II during the peroxidase cycle makes the LPO tetrapyrrole ring more susceptible to a direct HOCl attack. We propose that HOCl mediated cleavage of the tetrapyrrole moiety occurs through a radical based mechanism, as shown in our previous studies [36], [37]. Alternatively, the heme moiety of Compound II, in the presence of HOCl, could be destroyed through the formation of LPO-Fe(III)-OO^−^ radical [55]. Under these circumstances, the formation rate of the Compound II is comparable or slower that the decay of this intermediate, therefore the buildup of the LPO-Fe(III)-OO^−^ radical cannot be seen, and the conversion of Compound II to LPO-Fe(III)-OO^−^ is the rate limiting step and occurs independently of the HOCl concentration. LPO heme destruction is also confirmed by HPLC analysis, which revealed that at least three fluorescent heme degradation products were observed. Similar fluorescent bands were also observed when oxy-Hb, hematin, MPO, as well as protoporphyrin IX were treated with increasing concentrations of HOCl [36], [37]. Collectively, this work demonstrates the ability of HOCl to modulate heme destruction through oxidative cleavage of one or more carbon-methene bridges of the tetrapyrrole moiety. We suggest that this phenomenon may partially elucidate the significant role played by HOCl in pathological conditions.

**Figure 9:**
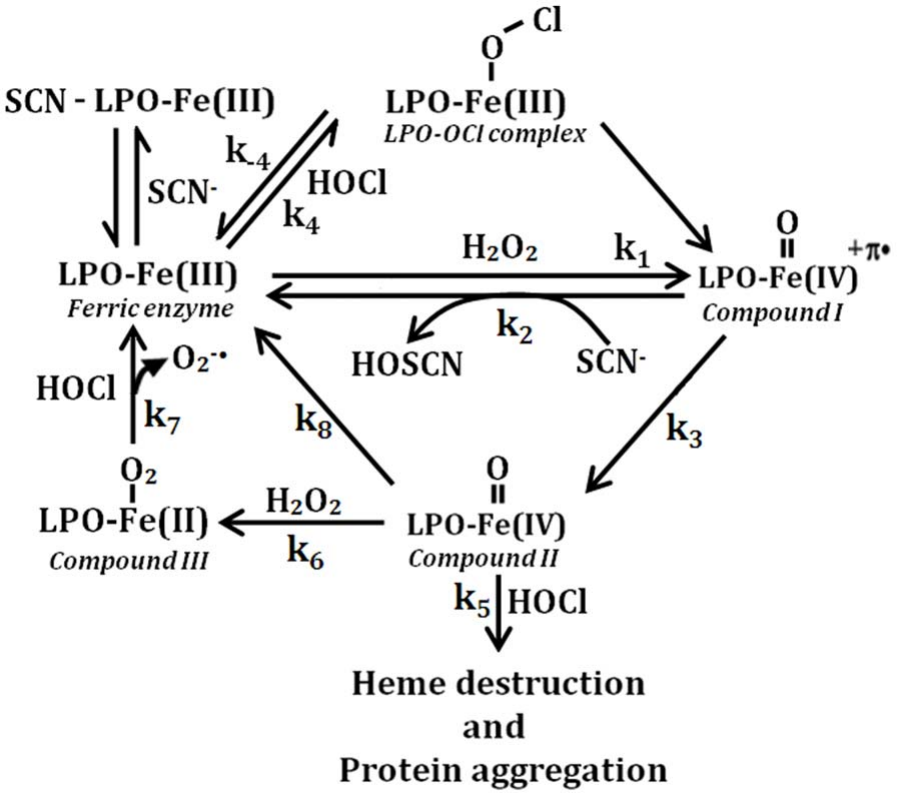
A general kinetic scheme showing the influence of HOCl on the LPO catalytic cycle.

HOCl also mediates the heme destruction of LPO-Fe(II)-O_2_ complex through a mechanism that initially involves heme oxidation, but otherwise the kinetics of heme destruction was almost similar to those obtained for LPO-Fe(III) with HOCl. Under these circumstances the oxidized form of the enzyme can no longer bind oxygen but it can bind the OCl^−^ molecule. Based on our kinetic model illustrated in Fig. 9 (see also Table 1, for the rate constants of each step), the three main pathways that lead to the formation of LPO Compound III are: the direct reaction between O_2_ and superoxide with LPO-Fe(II) and LPO-Fe(III), respectively, or the addition of a slight molar excess of H_2_O_2_ to LPO-Fe(III). The role of LPO Compound III in biological systems is still unclear. The reverse rate constant (k_off_) of LPO-Fe(II)-O_2_ binding is significantly higher when compared with that of other hemoproteins [16]. Several factors and conditions may account for the high dissociation rate in LPO-Fe(II)-O_2_, including: the positive *trans* effect contributed by the peroximal ligand, the heme pocket microenvironment, and the geometry of Fe-O_2_ linkage. This notion is consistent with earlier resonance Raman spectroscopy studies, which showed that the υ(Fe-O_2_) frequency for LPO was, to a large extent, lower than those reported for related and relevant hemoprotein model compounds [56] We believe that the high dissociation rate of LPO-Fe(II)-O_2_ complex [16] is a key feature that drives rapid oxidation and decomposition of the enzyme-O_2_ complex, and promotes generation of ligand-free LPO-Fe(III). Previously, we have shown that the formation of an LPO-Fe-O_2_ complex intermediate in the catalytic mechanism of the enzyme [16]. As such, low OCl^−^ concentration might represent an alternative pathway, whose biological function is to destabilize LPO Compound III, an inactive form of the enzyme, and restore its catalytic activity and rejoin the peroxidase cycle after the removal of unwanted HOCl.

**Table 1:** Rates for the reactions depicted in the kinetic model in Fig. 9.

Step	Rate	References
k_1_	11 µm^−1^ s^−1^	[47]
k_2_	200 µm^−1^ s^−1^	[47]
k_3_	0.014 µm^−1^ s^−1^	Present study
k_4_	12.1 µm^−1^ s^−1^	Present study
k_−4_	18 s^−1^	Present study
k_5_	1×10^−4^ µm^−1^ s^−1^	Present study
k_6_	2. 2×10^−4^ µm^−1^ s^−1^	[86]
k_7_	4×10^−4^ µm^−1^ s^−1^	Present study
k_8_	0.0005 s^−1^	[12]

A reducing and denaturing SDS-PAGE showed different degrees of protein aggregation as a function of HOCl concentration. The degree of LPO protein aggregation induced by HOCl was much less than when hemoglobin/apohemoglobin or catalase was treated with HOCl under identical experimental conditions. In addition, globin aggregation in hemoglobin occurred independently of iron presence. Indeed, previous studies by Chapman *et. al*. [57]. Utilizing extensive biochemical and mass-spectrometric studies have shown that as a result of exposure to HOCl, apohemoglobin undergoes aggregation and produces a regular series of high molecular weight oligomers [57]. This change in the protein structure in hemoglobin was facilitated by the formation of protein carbonyls and possibly chloramines, along with methionine oxidation, which altered the protein folding and, subsequently, the secondary/tertiary structure of the protein. The process of aggregation was due to non-covalent interaction between the exposed hydrophobic areas on neighboring molecules that associate to form dimers and higher-molecular mass aggregates [57]. Recent studies with intact hemoglobin have shown similar patterns of protein aggregation in reducing SDS PAGE, except for a distinct dimer band we observed, even at higher HOCl-Hb ratios [36]. This process could lead to the formation of aggregated proteins at sites of inflammation where MPO activity and subsequently HOCl generation is enhanced, and may contribute to tissue injury. Aggregated proteins are formed during aging [58], in diabetes [59] and in neurodegenerative diseases, including Creutzfeldt–Jacob disease, Huntington's disease, Alzheimer's disease and Parkinson's disease [60], [61].

LPO Compound I preferentially catalyzes the 2-electron oxidation of SCN^−^, generating the corresponding HOSCN. LPO Compound I may also oxidize SCN^−^ through a two sequential one-electron steps, forming Compound II and LPO-Fe(III), respectively [62]. The ability of both LPO Compounds I and II to employ SCN^−^ as a 1e^−^ substrate prevents LPO heme destruction mediated by HOCl, and influences the nature of the end products of the SCN^−^ oxidation reaction. Our results clearly showed that the protection of HOCl mediated LPO heme degradation require a ratio of at least 4∶1 HOCl∶SCN^−^ (Fig. 5). One electron oxidation of SCN^−^ yields unstable thiocyanate radical, which then dimerizes to generate a labile short-lived intermediate, thiocyanogen (SCN)_2_ [63], [64]. The resulting (SCN)_2_ is rapidly hydrolyzed to generate either HOSCN/OSCN^−^ or CN^−^ without the formation of HOSCN as an end product [61], [63], [65], [66]. Alternatively, SCN^−^ can react directly with HOCl to generate HOSCN [67]. Taken together, these studies suggest that LPO may serve as a catalytic sink for HOCl, regulating its bioavailability and function.

HOCl-mediated LPO heme destruction events are likely to occur extracellularly. LPO is secreted from the goblet cells and submucosal glands which is a major constituent of the mucus and is very important constituent for maintaining anti-infective properties of airway epithelium [68]. It is likely that MPO is independently secreted into sites of inflammation from phagocytic cells. In the airway, we believe that there is a balance between HOCl and SCN^−^. Disturbance of this balance allows LPO to serve as a source of free iron. The normal SCN^−^ concentration in the plasma is ranging from 20 to 120 µM, while in airways secretion it is about 460 µM [69], [70]. But under certain pathological conditions such as cystic fibrosis, the level of SCN^−^ is known to be, as low as, 0.5 µM which disrupts the microbicidal function of LPO [71]–[73]. Direct measurement of HOCl in the airways has not been done and would be difficult since HOCl is very labile and reactive. Using computational modeling the level of HOCl generated from activated neutrophils has been estimated to be 150–425 µM HOCl/hour and at sites of inflammation it has been estimated to reach, as high as, 5 mM [74]. During airway inflammation there is increased neutrophil influx and the neutrophil count in the airway increases from 34.1×10^3^/mL to 115.7×10^3^/mL [73]. Also the MPO protein level increases to 4.68 nM from 0.12 nM [73]. This increased MPO leads to an increase in HOCl production along with its subsequent damage to the biomolecules. Indeed Van der vliet *et. al.,* have shown that in cystic fibrosis, MPO is the chief mediator of oxidative damage within the respiratory tract [75]. They also suggested that the localized MPO concentration could reach around 0.5–10 µM with an estimated HOCl concentration of 760 µM [71]. Previous studies have demonstrated that the airways of cystic fibrosis patients contain increased amount of total iron and the iron-binding protein ferritin [44], [76]. This may explain why increased polymorpho neutrophil (PMN) phagocytosis cannot prevent *Pseudomonas aeruginosa* infection [44]. Increase in PMN influx will lead to an increase in HOCl and increase free iron which will accelerate *P. aeruginosa* growth. In related study, Agbai has shown that high SCN^−^ diets give rise to low incidence of sickle cell anemia in Africans, while SCN^−^ deficient American meals cause increase number of cases of the disease in African-American population [77].

We have obtained significant data demonstrating that HOCl can mediate hemoproteins heme destruction and subsequent liberate of free iron in complex biological systems such as intact red blood cells, despite their extensive content of antioxidant such as GSH/glutathione reductase, and catalase [36]. These antioxidant systems have been previously shown to be potent targets of HOCl consumption. We are currently examining the role of HOCl in destroying the RBC of sickle cell patients, a condition known to be associated with higher plasma complement activation and higher MPO levels, (Maitra & Abu-Soud, unpublished data). Since the chemistry between heme and HOCl that we have reported in this study is similar to that which occurs when hemoglobin react with HOCl, this phenomenon may occur in complex biological systems.

In summary, HOCl levels and LPO activity are apparently coupled through complex interdependent pathways. The biological consequences of HOCl-peroxidase interactions may have broad implication for the regulation of local inflammatory, infectious, and cardiovascular events *in vivo*. Increased HOCl levels, the deficiency of potent HOCl scavengers such as taurine, glutathione, and lycopene [78]–[80], or the deficiency of the natural enzyme substrate, SCN^−^, may contribute to infection and inflammation by increasing catalytically active free iron levels and enhancing bacterial development.

## Materials and Methods

### Materials

All materials used were of highest purity grade and used without further purification. Sodium hypochlorite (NaOCl), ammonium acetate (CH_3_COONH_3_), ferrozine, L-methionine, ascorbic acid, methanol and trifluoroacetic acid (TFA) - HPLC grade, were obtained from Sigma Aldrich (St. Louis, MO, USA). HPLC grade acetonitrile (CH_3_CN) was obtained from EMD Chemicals Inc. (Gibbstown, NJ, USA). Bovine LPO was obtained from Worthington Bio-Chemistry Corp. (Lakewood, NJ, USA) and used with further purification by sepharose column. Purity was confirmed by demonstrating a RZ (Reinheitszahl) of >0.78 (A_415_/A_280_), as well as SDS-PAGE analysis. LPO concentration was determined spectrophotometrically by utilizing an extinction coefficient of 112,000 M^−1^ cm^−1^ at 412 nm [81].

### Absorbance Measurements

Absorbance spectra were recorded using a Cary 100 Bio UV–visible spectrophotometer, at 25°C, pH 7.0. Experiments were performed in a 1 mL phosphate buffer solution supplemented with fixed amount of LPO (1.5 µM) and increasing concentrations of HOCl (0, 25, 50, 100, 150, 175, 200, and 300 µM). To accomplish that, LPO and HOCl were mixed in the cuvette, and then absorbance changes were recorded from 300 to 700 nm.

### Rapid Kinetic Measurements

Kinetic measurements of HOCl-mediated LPO heme destruction were performed using a dual syringe stopped-flow instrument obtained from Hi-Tech, Ltd. (Model SF-61). Measurements were carried out under an aerobic atmosphere at 10°C following rapid mixing of equal volumes of a buffer solution containing a fixed amount of LPO (3 µM final) and a buffer solution containing increasing concentration of HOCl. The time course of the absorbance change was fitted to a single-exponential, (Y = 1−e^−kt^), or a double-exponential (Y = Ae^−k1t^+Be^−k2t^) function as indicated. Signal-to-noise ratios for all kinetic analyses were improved by averaging at least six to eight individual traces. In some experiments, the stopped-flow instrument was attached to a rapid scanning diode array device (Hi-Tech) designed to collect multiple numbers of complete spectra (200–800 nm) at specific time ranges. The detector was automatically calibrated relative to a holmium oxide filter, as it has spectral peaks at 360.8, 418.5, 446.0, 453.4, 460.4, 536.4, and 637.5 nm, which were used by the software to correctly align pixel positions with wavelength.

### High Performance Liquid Chromatography (HPLC) analysis

HPLC analyses were carried out using a Shimadzu HPLC system equipped with a SCL-10A system controller, with a binary pump solvent delivery (LC-10 AD) module and a SIL-10AD auto-injector connected to a SPD-M10A diode array detector (DAD) and a RF-10A XL fluorescence detector. Alltech 5 µm particle size, 4.6×150 mm reverse-phase octadecylsilica (C18) HPLC column was used. The photodiode array detector was set at 400 nm and the fluorescent detector was set at excitation 321 nm and emission 465 nm to monitor the chromatogram. The column was eluted at a flow rate of 1.0 mL/min with linear gradients of solvents A and B (A, 0.1% TFA in water; B, 0.1% TFA in 80% acetonitrile). The solvent gradient was as follows: 0 to 10 min, 55–65%B; 10 to 14 min, 65–90% B; then the buffer B composition dropped down to 55% within 14 to 24 min. After treatment of LPO (1.5 µM) with HOCl (0 to 400 µM) for 2 hours, the reaction was stoped with 5 molar excess of methionine, and 500 µL of the reaction mixture was diluted with 500 µL of injection solvent (55% B and 45% A) and 50 µL were injected. After the end of the run, the system was equilibrated with 45% solvent A. Each sample was analyzed in triplicate. After treatment with HOCl, the reaction mixture was filtered through an Amicon Ultra-15 centrifugal filter unit with Ultracel-10 membrane (from Millipore) with a 3-kDa cut-off by centrifuging at 14,000 *g* for 30 min at 4°C.

### Free iron analysis

Free iron release was measured colorimetrically by using the ferrozine-based assay, following a slight modification of a published method [82]. To 100 µL of the sample (LPO-HOCl reaction mixture), 100 µL of ascorbic acid (100 mM) were added. After 5 minutes of incubation at room temperature, 50 µL of ammonium acetate (16%) and the same volume of ferrozine (16 mM) were added to the mixture and mixed well. Again, after 5 minutes of incubation at room temperature, the absorbance was measured at 562 nm. A standard curve prepared by using ammonium Fe(III) sulfate was used for the calculation of free iron concentration. Final concentrations of the additives are as follows, ascorbic acid-33.33 µM, ammonium acetate 5.3%, and ferrozine 5.3 µM.

### SDS-PAGE

Samples of LPO (1.5 µM) were mixed with increasing HOCl concentrations (0 to 300 µM) and incubated for two hours at room temperature. After reaction completion, methionine (5-fold of the final HOCl concentration) were added to eliminate excess HOCl, and 10 µg of LPO (from the reaction mixtures) were incubated with Laemmli buffer [83] containing 63 mM Tris–HCl (pH 6.8), 2% (w/v) SDS, 10% (w/v) glycerol, 0.0025% (w/v) bromophenol blue, and 10% (v/v) 2-mercaptoethanol. The samples were boiled for 5 minutes at 100°C before loading, and gel electrophoresis was performed for 2 h at a constant voltage of 60 V on 4–12% gradient gels. Three sets of gels were run at room temperature on different days. The gels were then stained with Coomassie blue for 24 hours, destained in methanol/acetic acid to remove background and digitalized. The band intensity of the 77 KDa, corresponding to LPO was quantified using ImageJ (NIH) software and the intensity of the control (untreated LPO) was compared to that of HOCl treated samples. Further the ratio of the band intensities between LPO 77 KDa and the higher molecular weight aggregates were also plotted as a function of HOCl concentration. Relative amounts of protein were estimated by densitometric analysis of the images using Image J software from the NIH [32].

### Solution preparation

HOCl preparation - HOCl was prepared following a slight modification of a published method [84]. Briefly, a stock solution of HOCl was prepared by adding 1 ml NaOCl solution to 40 ml of 154 mM NaCl, and the pH was adjusted to around 3 by adding HCl. The concentration of active total chlorine species in solution expressed as [HOCl]_T_ (where [HOCl]_T_ = [HOCl]+[Cl_2_]+[Cl_3_^−^]+[OCl^−^]) in 154 mM NaCl was determined by converting all the active chlorine species to OCl^−^ by adding a bolus of 40 µL of 5 M NaOH and measuring the concentration of OCl^−^. The concentration of OCl^−^ was determined spectrophotometrically at 292 nm (ε = 362 M^−1^ cm^−1^) [85]. As HOCl is unstable, the stock solution was freshly prepared on a daily basis, stored on ice, and used within one hour of preparation. For further experiments, dilutions were made from the stock solution using 200 mM phosphate buffer pH 7, to give working solutions of lower HOCl concentration.
